# Intravenous Glutamine Administration Improves Glucose Tolerance and Attenuates the Inflammatory Response in Diet-Induced Obese Mice after Sleeve Gastrectomy

**DOI:** 10.3390/nu12103192

**Published:** 2020-10-19

**Authors:** Chiu-Li Yeh, Po-Jen Yang, Po-Chu Lee, Jin-Ming Wu, Po-Da Chen, Chun-Chieh Huang, Sung-Ling Yeh, Ming-Tsan Lin

**Affiliations:** 1 School of Nutrition and Health Sciences, College of Nutrition, Taipei Medical University, Taipei 11031, Taiwan; clyeh@tmu.edu.tw (C.-L.Y.); sangling@tmu.edu.tw (S.-L.Y.); 2 Nutrition Research Center, Taipei Medical University Hospital, Taipei 11031, Taiwan; 3 Research Center of Geriatric Nutrition, College of Nutrition, Taipei Medical University, Taipei 11031, Taiwan; 4 Department of Surgery, National Taiwan University Hospital, College of Medicine, National Taiwan University, Taipei 10002, Taiwan; paulpjyang@gmail.com (P.-J.Y.); d97421103@ntu.edu.tw (P.-C.L.); wujm0531@ntu.edu.tw (J.-M.W.); podachen@gmail.com (P.-D.C.); yujiahcc@yahoo.com.tw (C.-C.H.); 5 Department of Surgery, National Taiwan University Hospital Hsin-Chu Biomedical Science Park Branch, Hsin-Chu County 302, Taiwan; 6 Department of Surgery, National Taiwan University Hospital Hsin-Chu Branch, Hsin-Chu County 30059, Taiwan

**Keywords:** obesity, sleeve gastrectomy, glutamine, glucose tolerance, hepatic proteomic profiles

## Abstract

Obesity is a health problem associated with many metabolic disorders. Weight reduction can effectively alleviate obesity-associated complications. Sleeve gastrectomy is a commonly used bariatric surgery and is considered safe and effective for improving outcomes. Glutamine (GLN) is an amino acid with anti-oxidative and anti-inflammatory properties. This study used a mouse model of sleeve gastrectomy to investigate the impacts of intravenous GLN administration on glucose tolerance and adipocyte inflammation short-term after surgery. C57BL6 male mice were divided into normal control (NC) and high-fat diet groups. The high-fat diet provided 60% of energy from fat for 10 weeks to induce obesity. Mice fed the high-fat diet were then assigned to a sham (SH) or sleeve gastrectomy with saline (S) or GLN (G) groups. The S group was intravenously injected with saline, while the G group was administered GLN (0.75 g/kg body weight) via a tail vein postoperatively. Mice in the experimental groups were sacrificed on day 1 or 3 after the surgery. Results showed that obesity resulted in fat accumulation, elevated glucose levels, and adipokines production. Sleeve gastrectomy aggravated expressions of inflammatory cytokine and macrophage infiltration markers, cluster of differentiation 68 (CD68), epidermal growth factor-like module-containing mucin-like hormone receptor-like 1 (EMR-1), and macrophage chemoattractant protein-1, in adipose tissues. Treatment of obese mice with GLN downregulated hepatic proteomic profiles associated with the gluconeogenesis pathway and improved glucose tolerance. Moreover, macrophage infiltration and adipose tissue inflammation were attenuated after the sleeve gastrectomy. These findings imply that postoperative intravenous GLN administration may improve glucose tolerance and attenuate inflammation shortly after the bariatric surgery in subjects with obesity.

## 1. Introduction

Obesity is an important public health issue worldwide. According to a report from three consecutive waves of the Nutrition and Health Surveys in Taiwan (NAHSIT), prevalence of morbid obesity and obesity increased sharply from 1993–1996 and 2013–2014 [1]. Obesity is a condition of low-grade systemic inflammation. Immune cells infiltrate into enlarged adipocytes leading to persistent proinflammatory mediator production, which is considered the sole mechanism [2]. Excessive fat mass accumulation and adipose tissue inflammation are positively correlated with insulin resistance (IR), glucose intolerance, and other associated metabolic dysfunctions [2].

Epidemiological studies showed that obesity is associated with increased risks of many metabolic disorders, such as cardiovascular diseases, hypertension, type 2 diabetes, stroke, etc. [3], and mortality is also increased [4]. Weight reduction can effectively alleviate multiple obesity-related complications [5]. However, conservative treatment of obesity including diet restriction, behavior modification, and pharmacological methods are ineffective for long-term weight control [6,7]. Body weight (BW) rebound frequently occurs, especially in subjects with morbid obesity. Sleeve gastrectomy is a bariatric surgery commonly used in patients with morbid obesity and is considered a safe and effective operation to lose weight and attenuate comorbidities [8,9].

Glutamine (GLN) is an amino acid with immunomodulatory properties. Previous studies showed that GLN administration had favorable effects on catabolic conditions through its anti-oxidative and anti-inflammation properties, and it exerts more-balanced immune cell regulation [10,11,12]. A clinical study reported that parenteral GLN supplementation during the postoperative period for gastrectomy patients reduced the rate of infectious complications, improved immune functions, and shortened hospital stays [13]. Our recent study demonstrated that intravenous GLN administration increases serum albumin levels and benefits patients receiving gastric cancer surgery [14]. A study performed by Abboud et al. found that a high-fat diet supplemented with GLN feeding for 4 weeks improved insulin’s action and reversed the defects in hepatic glucose metabolism in diet-induced obesity (DIO) rats [15]. Moreover, the waist circumference and serum insulin levels in subjects with obesity were found to be reduced after 2 weeks of GLN supplementation [15]. Although GLN seems to have favorable effects on obesity and postoperative conditions, we are unaware of any study investigating impacts of GLN on metabolic alterations and inflammatory reactions in response to bariatric surgery in subjects with obesity. Moreover, no study has applied proteomic techniques to address the effects of GLN after sleeve gastrectomy. Since the liver is the main organ for regulating glucose homeostasis under stressed conditions, profiling the hepatic proteomic response to GLN after sleeve gastrectomy is worth evaluation. In this study, we used a mouse model to mimic sleeve gastrectomy performed in humans with morbid obesity to investigate the effects of intravenous GLN administration on inflammatory responses and liver proteomic profiles associated with glucose metabolism 1 d and 3 d after gastrectomy. We hypothesized that postoperative GLN administration after sleeve gastrectomy would improve glucose tolerance and alleviate adipose tissue inflammation in DIO mice shortly after the surgery.

## 2. Materials and Methods

### 2.1. Animals

Male C57BL/6 mice (5 weeks old, weighing 18~20 g, *n* = 56) were used in the study. All mice were kept under controlled temperature (21 ± 2 °C) and relative humidity (50%~55%) conditions with a 12-h light-dark cycle in the Laboratory Animal Center at Taipei Medical University (TMU, Taipei, Taiwan). A standard rodent chow diet (Purina no. 5001, Fort Worth, TX, USA) and water were provided ad libitum. Care of laboratory animals was in full compliance with the Guide for the Care and Use of Laboratory Animals (National Research Council, 1996). Experimental protocols were approved by the Animal Care and Use Committee at TMU (Laboratory Animal Center-2019-0082).

### 2.2. Experimental Design

Mice were randomly assigned to a normal control (NC, *n* = 8) group and a high-fat diet (HF, *n* = 48) group. Mice in the NC group were fed a rodent chow diet for 10 weeks, while mice in the HF group were provided a high-fat diet (Research Diets, Inc., New Brunswick, NJ, USA), which provided 60% of kcal as fat for 10 weeks. The high-fat diet composition is listed in Table 1. Then mice in the HF group were divided into a high-fat sham (SH, *n* = 8) group, high-fat gastrectomy group with saline injection (S, *n* = 20), and high-fat gastrectomy group with GLN administration (G, *n* = 20). Mice in the SH group were subjected to a laparotomy operation but without a gastrectomy. The two gastrectomy groups were subjected to sleeve gastrectomy surgery as previously described [16]. Briefly, mice were anesthetized with an intraperitoneal injection of Zoletil (25 mg/kg BW; Virbac, Carros, France) and Rompun (10 mg/kg BW; Bayer, Leverkusen, Germany). An animal’s abdomen was shaved and aseptically prepared. A midline incision was performed at the xiphoid process and extended to the lower abdominal providing exposure of the entire splanchnic bed. The sleeve gastrectomy was done in the same way as in patients. The lower portion of the esophagus and entire stomach were exposed. The gastrosplenic ligament and vessels were ligated and cut. After the stomach was completely mobilized and isolated, a clamp was placed on the stomach 0.8 cm to the lesser curvatura ventriculi minor curve of the stomach. The transection extended from the uppermost part of the forestomach to the lower portion of the greater curvature which were excised and removed. The remaining stomach was sutured using uninterrupted 7-0 nylon monofilament sutures. All mice performed the operation were given the antibiotic ertapenem (75 mg/kg BW, Invanz, Merck, Whitehouse Station, NJ, USA) at the operative site. Saline (40 mL/kg) containing the analgesic buprenorphine (0.2 mg/kg BW) was provided for resuscitation and pain control for 1 or 2 consecutive days. All of the gastrectomy surgeries were performed by the same person to ensure consistency. The two gastrectomy groups (S and G) were further subdivided into two groups according to the sacrifice schedule on day 1 (1S and 1G) or 3 (3S and 3G) after the operation. One hour after surgery, the 1S group was injected with saline, while the 1G group was given a single dose of alanyl-GLN dipeptide (Dipeptiven, Fresenius-Kabi, Homburg, Germany), which provided 0.75 g GLN/kg BW intravenously via a tail vein. Mice sacrificed on day 3 were provided another dose of saline (3S) or GLN (3G) injection on day 2 postoperatively (24 h after the first dose of saline or GLN) according to the respective grouping. The dosage of GLN used here was shown to attenuate inflammation and modulate immune response during catabolic state [17]. On the day of sacrifice, mice were euthanized by cardiac puncture. Blood samples were collected in tubes containing heparin. Plasma samples were separated from whole blood by centrifugation at 1500× *g* and 4 °C for 10 min. The peritoneum was opened and irrigated with 5 mL/100 g BW of saline to obtain peritoneal lavage fluid (PLF). The plasma and PLF were stored at −80 °C. Epididymal tissues were weighed. The epididymal and liver tissues were frozen in liquid nitrogen and stored at −80 °C for further analysis.

### 2.3. Measurements of Plasma Biochemical Markers and Adipokines

Liver function markers, including aspartate aminotransferase (AST) and alanine aminotransferase (ALT), were measured using commercial kits (BioVision, Milpitas, CA, USA). Fasting plasma glucose was measured by the glucose oxidase method, and insulin was analyzed by an enzyme-linked immunosorbent assay (ELISA) kit (R&D Systems, Minneapolis, MN, USA) following the manufacturer’s instructions.

### 2.4. Inflammatory Cytokine Concentrations in PLF

Local inflammatory markers, including interleukin (IL)-1β, IL-6, and tumor necrosis factor (TNF)-α were analyzed using ELISAs in a microtiter plate. Antibodies specific for mouse IL-1β, IL-6, and TNF-α were first coated onto the wells of microtiter strips provided by the manufacturer (eBioscience, San Diego, CA, USA), incubated with the samples, and then developed with reagents. The absorbance of each well was measured with a spectrophotometer.

### 2.5. Messenger (m)RNA Extraction and a Real-Time Reverse-Transcription (RT) Quantitative Polymerase Chain Reaction (qPCR) Analysis

Total RNA was isolated from homogenized epididymal tissues using the TRIzol reagent (Invitrogen, Carlsbad, CA, USA) method. The RNA pellet was dissolved in RNase-free water. The total RNA solution was stored at −80 °C for the subsequent assay. The RNA concentration was determined and quantified by measuring absorbances at 260 and 280 nm on a spectrophotometer. Complementary (c)DNA was synthesized from total RNA using a RevertAid™ first-strand cDNA synthesis kit (Fermentas, Vilnius, Lithuania) according to standard protocols. RT was carried out by subsequent incubation for 5 min at 65 °C, 60 min at 42 °C, and 5 min at 70 °C. cDNA was stored at −80 °C until being used. Specific mRNA genes were amplified by a real-time RT-PCR using the 7300 Real-Time PCR System (Applied Biosystems, Foster City, CA, USA) with SYBR Green I as the detection format. The genes analyzed included macrophage infiltration markers (cluster of differentiation 68 (CD68), epidermal growth factor-like module-containing mucin-like hormone receptor-like (EMR)-1, and monocyte chemoattractant protein (MCP)-1) and inflammatory cytokines (IL-1β, IL-6, and TNF-α). Primers used in this study are listed in Table 2. Primers were purchased from Mission Biotech (Taipei, Taiwan) based on deposited cDNA sequences (GenBank database, National Center for Biotechnology Information). Amplification was carried out in a total volume of 25 µL containing 1× Power SYBR Green PCR Master Mix (Applied Biosystems), 400 nM of each primer, and 100 ng of cDNA. The reaction was performed as one cycle of 2 min at 50 °C and 10 min at 95 °C, followed by 40 cycles of 15 s at 95 °C and 1 min at 60 °C, with a final dissociation curve (DC) analysis. Expression levels were quantified in duplicate by means of a real-time RT-PCR. Cycle threshold (CT) values of genes of interest were normalized to mice β-actin and were used to calculate the relative quantity of mRNA expression [18].

### 2.6. Proteomics Sample Preparation

Twenty micrograms of freeze-dried liver proteins were solubilized in 10 µL of 2 mM dithioerythritol/8 M urea in 25 mM ABC (NH_4_HCO_3_, ammonium bicarbonate buffer at pH 8.0) for disulfide bond reduction at 37 °C for 45 min, and 10 µL of 20 mM iodoacetamide in 25 mM ABC was added for alkylation for 45 min at room temperature in the dark. Trypsin was added and incubated at 37 °C overnight. The digestion reaction was stopped by adding 10 µL of 1% formic acid (FA). Digested peptides were desalted using a C18 Zip-Tip and eluted with 50% acetonitrile (ACN) and 0.1% FA. The eluate was freeze-dried and solubilized in 100 µL of 0.1% FA in 95:5 water/ACN before ultra-performance liquid chromatographic quadrupole orthogonal time of flight mass spectroscopic (UPLC-Q-TOF MS) analysis.

### 2.7. UPLC-Q-TOF MS Analysis

Five microliters of desalted peptides were analyzed in triplicate in an XBridge BEH130 C18 5-µm desalting/trap column on-line with a BEH300 C18 1.7-µm nanoUPLC analytical capillary column (100 µm × 100 mm) on an ACQUITY nanoUPLC-LC system interfaced with a nano source to a SYNAPT G2 Q-TOF MS (Waters MS Technologies, Manchester, UK). The entire length of the LC run was 120 min starting with a gradient of ACN/0.1 FA (1% to 40%) from 2 to 70 min followed by an 85% ACN wash and re-equilibration. Data were acquired in the V-positive mode with Glu-Fib as the calibrant (*m*/*z* 785.8426) and lock mass. MS and MS/MS data were recorded in the MS^E^ mode (MS1 scan every 0.6 s at 10,000 Firmware Hub resolution and MS/MS fragmentation of all ions every 0.6 s). Acquisition of all data was controlled by Waters MassLynx v4.1 software. MS^E^ runs were also analyzed by Protein Lynx Global Server 3.0.3 software for validation of protein hits and the peptide coverage map.

### 2.8. Differential Label-Free Quantitative and Qualitative Proteomics

Progenesis QI for Proteomics version 4.2 (Nonlinear Dynamics, Newcastle, UK) was used to analyze the different MS^E^ runs in triplicate. Samples were compared according to their exact mass versus the retention time ratio after normalization to total proteins. Peptides were identified using the Progenesis QI for proteomics internal MSe search engine based on ProteinLynx Global Server 3.0.3 and the human protein FASTA database created according to the respective UniProt sequences. Progenesis was quantified by the built-in Hi-3 algorithm from Waters which allowed, after Apex 3D peptide identification of the reference protein digest, the quantification of any protein based on their three most abundant peptides. The fixed modification was carbamidomethyl cysteine (+57.02 Da), and other variable modifications were also accounted for in the search, such as deamidation (+0.98) and oxidation Multihundred-Watt (15.99). The protein normalization method chosen in Progenesis, as mentioned previously, was performed according to total proteins as well as absolute quantification.

### 2.9. Ingenuity Pathway Analysis (IPA) of the Proteomic Profiles in the Liver

Proteins identified and quantified by Progenesis QI with a 2-fold change in abundance (log2 ratio of >1.0 and <−1.0) were chosen and a *p* value cutoff of 0.05 was applied in the IPA (Ingenuity^®^ System, Redwood City, CA, USA. www.ingenuity.com). Protein data along with individual multiples of change were uploaded to IPA software for grouping interactions and analyzing canonical pathways. Matched proteins encoding genes identified pathways from the IPA Knowledge Base. Based on the IPA’s analysis, significant canonical pathways, biological functions, diseases, and interaction networks were algorithmically generated to predict the activation status of these processes.

### 2.10. Statistical Analyses

Data are expressed as the mean ± standard error of the mean (SEM). All analyses were conducted using GraphPad Prism 5 (GraphPad Software, La Jolla, CA, USA). Differences between the NC and high-fat sham (SH) groups were compared with a *t*-test. A one-way analysis of variance (ANOVA) followed by the Bonferroni post-hoc test was used to analyze differences among the SH and gastrectomy groups. A *p* value of <0.05 was considered statistically significant. ANOVA statistics were also calculated using the built-in Progenesis statistics module. Significance levels of associations between the dataset and canonical pathways were calculated by Fisher’s exact test, and *p* < 0.05 was accepted as a significant difference.

## 3. Results

### 3.1. BW and Epididymal Weight Changes after Feeding the High-Fat Diet for 10 Weeks

Initial BWs were similar between the NC and HF groups. After 10 weeks of feeding, the BW of the HF group was significantly higher than that of the NC group (NC 25.8 ± 0.8 g vs. HF 31.9 ± 1.6 g, *p* < 0.05). The epididymal fat weight of the HF group was also higher than that of the NC group (NC 0.45 ± 0.23 g vs. HF 1.58 ± 0.15 g, *p* < 0.05).

### 3.2. Plasma Adipokine, Glucose, and Insulin Levels after the High-Fat Diet Intervention

DIO resulted in elevated leptin and adiponectin concentrations. Glucose and insulin levels were also higher after 10 weeks of high-fat diet feeding (Table 3).

### 3.3. Effects of GLN on BW Change after a Gastrectomy

BW changes did not differ between the two gastrectomy groups 1 day after surgery (1S −1.77 ± 0.82 g vs. 1G −1.49 ± 0.97 g; *p* > 0.05). However, mice administered GLN had lost more weight than the saline group 3 days after the gastrectomy (3S −2.48 ± 0.33 g vs. 3G −3.62 ± 0.41 g; *p* < 0.05).

### 3.4. Effects of GLN on Plasma Biochemical Parameters in Obese Mice after a Gastrectomy

**Figure 1:**
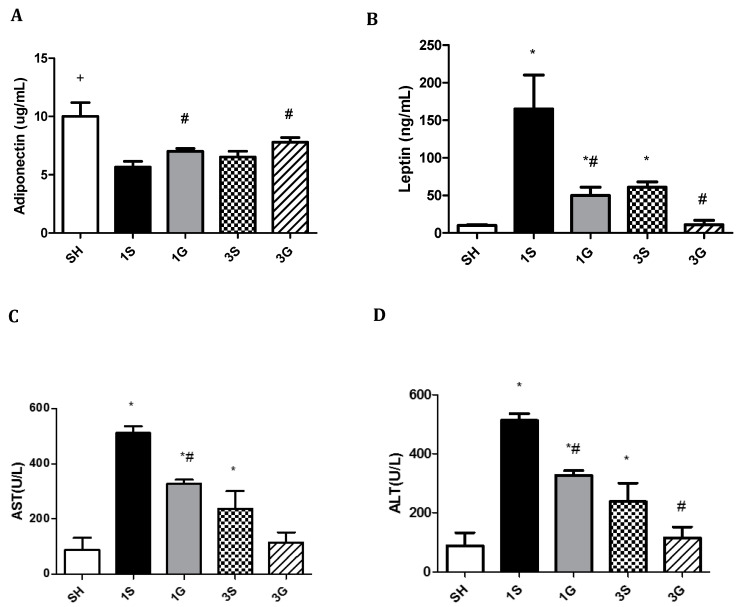
Plasma levels of (**A**) adiponectin, (**B**) leptin, (**C**) AST and (**D**) ALT among the sham group and experimental groups on days 1 and 3 after a sleeve gastrectomy. Data are presented as the mean ± SEM. AST, aspartate aminotransferase; ALT, alanine aminotransferase. SH, sham group; 1S, saline-injected group sacrificed 1 day after the gastrectomy; 1G, GLN-injected group sacrificed 1 day after the gastrectomy; 3S, saline group sacrificed 3 days after the gastrectomy; 3G, GLN-injected group sacrificed 3 days after the gastrectomy. *n* = 8 for each group. Differences among groups were analyzed by a one-way analysis of variance (ANOVA) followed by the Bonferroni post-hoc test. ^+^ Significantly differs from the gastrectomy groups. * Significantly differs from the SH group. ^#^ Significantly differs from the S group at the same time point (*p* < 0.05).

Compared to the SH and S groups, glucose and insulin concentrations were significantly lower in the G group on both days 1 and 3 after the gastrectomy. There were no differences in glucose or insulin levels between the SH and S groups after surgery (Table 4). Compared to the SH group, a sleeve gastrectomy resulted in decreased plasma adiponectin, while leptin, ALT, and AST concentrations increased several fold, especially on day 1 post-surgery. By day 3, the elevated levels of leptin, ALT, and AST had diminished in both gastrectomy groups. Compared to the S group, the G group had higher adiponectin, whereas leptin, ALT, and AST levels were lower on days 1 and 3 after the gastrectomy. There were no differences in levels of leptin, ALT, or AST between the SH and G groups 3 days after surgery (Figure 1).

### 3.5. Effects of GLN on Cytokine Levels in PLF

**Figure 2:**
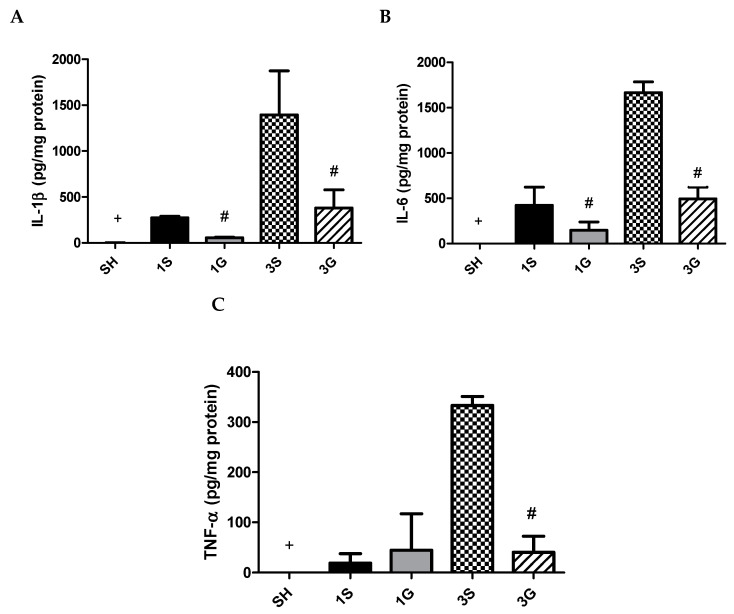
Concentrations of inflammatory cytokine (**A**) IL-1β, (**B**) IL-6 and (**C**) TNF-α in peritoneal lavage fluid. IL-1β, interleukin-1β; IL-6, interleukin-6; TNF-α, tumor necrosis factor-α. SH, sham group; 1S, saline-injected group sacrificed 1 day after the gastrectomy; 1G, GLN-injected group sacrificed 1 day after the gastrectomy; 3S, saline group sacrificed 3 days after the gastrectomy; 3G, GLN-injected group sacrificed 3 days after the gastrectomy. *n* = 8 for each group. Values are presented as the mean ± SEM. Differences among groups were analyzed by a one-way analysis of variance (ANOVA) followed by the Bonferroni post-hoc test. ^+^ Significantly differs from the SH group. ^#^ Significantly differs from the S group at the same time point (*p* < 0.05).

A gastrectomy resulted in increased IL-1β, IL-6, and TNF-α levels on day 1 in PLF. These inflammatory cytokines exhibited more-pronounced elevations on day 3 after surgery. Compared to the SH group, the G group had lower IL-1β and IL-6 at both time points and lower TNF-α on day 3 after the gastrectomy (Figure 2).

### 3.6. Expression of Macrophage Infiltration Markers and Inflammatory Mediator mRNAs in Epididymal Tissues after the Gastrectomy

**Figure 3:**
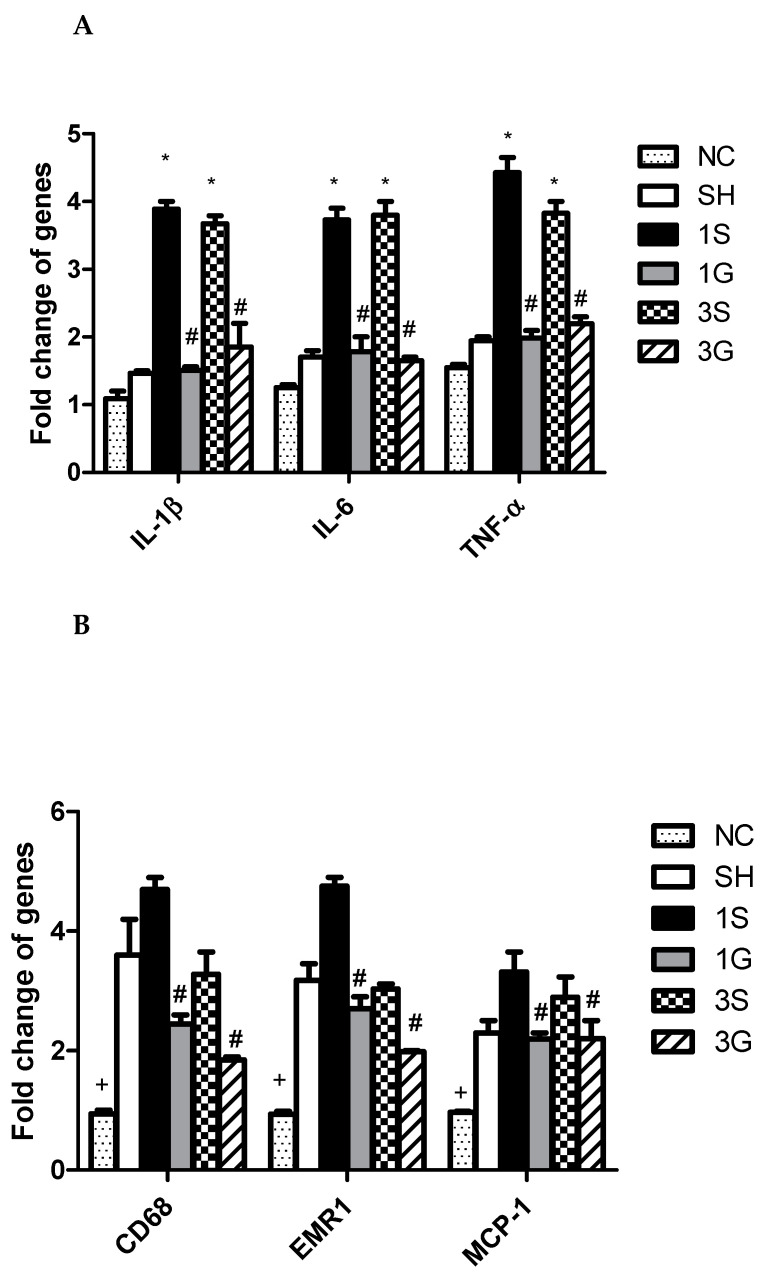
Messenger RNA expression of (**A**) inflammatory cytokines and (**B**) macrophage infiltration markers in epididymal fat tissues. IL-1β, interleukin-1β; IL-6, interleukin-6; TNF-α, tumor necrosis factor-α; EMR-1, epidermal growth factor-like module-containing mucin-like hormone receptor-like 1, MCP-1, monocyte chemoattractant protein-1. NC, normal control group (*n* = 6). SH, sham group; 1S, saline-injected group sacrificed 1 day after the gastrectomy; 1G, GLN-injected group sacrificed 1 day after the gastrectomy; 3S, saline group sacrificed 3 days after the gastrectomy; 3G, GLN-injected group sacrificed 3 days after the gastrectomy. *n* = 8 for the SH and gastrectomy groups. Values are presented as the mean ± SEM. Differences among groups were analyzed by a one-way analysis of variance (ANOVA) followed by the Bonferroni post-hoc test. ^+^ Significantly differs from the other groups. * Significantly differs from the NC and SH groups. ^#^ Significantly differs from the S group at the same time point (*p* < 0.05).

Compared to the normal control, obesity and gastrectomy resulted in significantly higher CD68, EMR-1, and MCP-1 gene expressions. However, there were no significant differences in expressions of IL-1β, IL-6, or TNF-α between the NC and SH groups. The inflammatory cytokines of IL-1β, IL-6, and TNF-α were all elevated after the gastrectomy. The macrophage infiltration markers of CD68, EMR-1, and MCP-1, and the inflammatory cytokines were significantly lower in the G groups compared to the S groups after the operation (Figure 3). 

### 3.7. Hepatic Proteomic Profiles Analyzed by the IPA

**Figure 4:**
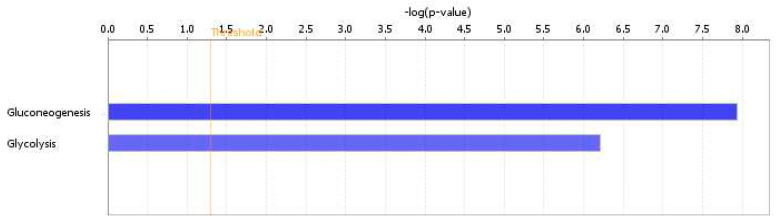
Comparison of canonical pathways between the 3S and 3G groups. Only two different pathways were shown to be related to glucose metabolism. The vertical line indicates a threshold of *p* < 0.05. Significant canonical pathways were determined by an ingenuity system pathway analysis. The x-axis displays the negative log of the *p* value calculated by Fisher’s exact test. The gluconeogenesis pathway is shown in a deeper blue color, indicating greater significance than the glycolysis. *p* values of gluconeogenesis and glycolysis were 1.5 × 10^−8^ and 6.12 × 10^−7^, respectively.

There were 448 proteins identified by Progenesis QI, and 262 proteins showed significant differences between the dataset and the canonical pathways analyzed by the IPA system. We focused on pathways related to glucose metabolism. There were no differences in protein sets between the 1S and 1G groups, however, the 3S and 3G groups showed significantly different protein sets in glycolysis and gluconeogenesis. The *p* values of gluconeogenesis and glycolysis were 1.5 × 10^−8^ and 6.12 × 10^−7^, respectively (Figure 4). The hepatic proteomic profiles showed that there were seven enzymes in gluconeogenesis and four enzymes in glycolysis pathway which significantly differed between the 3G and 3S groups. The fold changes of proteins were presented as log_2_ 3S/3G. Positive numbers indicate that the 3S group had higher aldolase, fructose-bisphosphate B (ALDOB), enolase 1 (ENO1), fructose-bisphosphatase 1 (FBP1), malate dehydrogenase 1 (MDH1), pyruvate carboxylase (PC), glyceraldehyde-3-phosphate dehydrogenase (GAPDH), and triosephosphate isomerase 1 (TPI1) expressions than those of the 3G group (Table 5). The gluconeogenesis and glycolysis pathways have some enzymes in common; however, the enzymes unique for glycolysis, including hexokinase, phosphofructokinase, and pyruvate kinase, did not have statistical differences between the 3S and 3G groups and were not analyzed by IPA.

## 4. Discussion

Sleeve gastrectomy is the most commonly performed bariatric procedure worldwide [19]. In this study, a surgical procedure of sleeve gastrectomy was carried out in DIO mice. This animal model mimics bariatric surgery used in patients with obesity and allowed us to investigate the effects of a nutrient intervention on molecular mechanisms after surgery. This is the first study to investigate the effects of intravenous GLN administration on glucose homeostasis by identifying hepatic proteomic profiles after bariatric surgery. Moreover, the influence of GLN on adipocyte inflammatory responses was evaluated. This study reported differential proteomics of the GLN intervention after sleeve gastrectomy. The proteomic datasets were analyzed by an IPA, and significant glycolysis and gluconeogenesis pathways were identified between groups with and without GLN administration 3 d post-surgery. We focused on the metabolic changes at postoperative days 1 and 3, because despite there has strong evidence for the long-term efficacy and safety [19], the bariatric surgery itself may exacerbate the inflammation with increased oxidative stress [20]. Attenuation of the inflammatory response shortly after the surgery may have metabolic benefits afterward. The main findings showed that intravenous GLN administration downregulated the pathway of gluconeogenesis and attenuated adipose tissue inflammation in DIO mice shortly after the sleeve gastrectomy.

High-fat diet feeding for 10 weeks produced significant increases in weight gain, adiposity, glucose intolerance, and IR. These findings were consistent with previous reports in DIO mice [21,22]. Adipose tissues are recognized as a multifunctional organ. In addition to lipid storage, they secrete several hormones called adipokines, which participate in energy balance, lipid metabolism, vascular homeostasis, inflammation, acute-phase responses, etc. [23]. Among the adipokines, leptin and adiponectin are the most notable that are involved in immune modulation in obesity. Leptin promotes inflammatory responses in obesity [24], while adiponectin inhibits TNF-α production in macrophages and is considered to have anti-inflammatory properties [23]. In this study, high-fat diet feeding produced significant increases in leptin and adiponectin levels indicating enhanced adipokine secretion in response to adipose tissue accumulation.

Obesity is recognized as a chronic inflammatory state. IL-1β, IL-6, and TNF-α are cytokines secreted during an inflammatory response. CD68 is a protein highly expressed by macrophages of inflamed tissues [25]. EMR-1, also known as F4/80, is a well-known and widely used marker of murine macrophages [26]. MCP-1, a monocyte activating protein, is involved in recruiting leukocytes to sites of inflammation [27]. Higher plasma leptin levels accompanying epididymal inflammatory cytokines and macrophage-infiltration gene expressions after sleeve gastrectomy suggest systemic inflammation occurred after surgery. A previous animal study showed that glucose tolerance had improved 3 weeks after bariatric surgery [22]. Since this study focused on bariatric surgery-associated glucose metabolism and inflammation, biochemical parameters were analyzed shortly after the operation. Therefore, weight loss and alterations in plasma glucose and insulin were not obvious when treated with saline. We also observed that obese mice performed gastrectomy resulted in higher liver transaminase levels. These results were consistent with previous clinical study, the authors reported a transient elevation of hepatic transaminases after gastric bypass in obese subjects. The ALT and AST levels peaked at 24 h and almost return to within baseline levels by 72 h. The mechanisms were found to be correlated with the operative time, the pressure of pneumoperitoneum, or musculoskeletal injury of the abdominal wall after open abdominal operations [28].

In this study, we found that DIO mice intravenously administered GLN after sleeve gastrectomy exhibited several favorable effects that were not observed in the saline groups. First, GLN administration improved glucose tolerance after the surgery. Hepatic proteomic profiles showed that GLN administration to DIO mice downregulated gluconeogenesis-specific enzyme expressions after sleeve gastrectomy. Gluconeogenesis and glycolysis are reciprocally regulated. Gluconeogenesis is a pathway that generates glucose from non-carbohydrate substrates, while glycolysis breaks down glucose into pyruvate to generate energy. These two pathways have some of the enzymes in common; however, there are irreversible steps to ensure that glycolysis and gluconeogenesis do not take place simultaneously in the same cell to a significant extent. Aldolase, enolase, GAPDH, and TPI1 are common enzymes in both gluconeogenesis and glycolysis, whereas MDH, FBP1, and PC are enzymes specifically involved in the process of gluconeogenesis. MDH and PC participate in the conversion of pyruvate to phosphoenolpyruvic acid, and FBP1 converts fructose-1,6-bisphosphate to fructose-6-P [29]. Our findings revealed that enzymes involved in gluconeogenesis were downregulated, whereas the process of glycolysis was not obviously changed when GLN was administered. Since inhibition of gluconeogenesis reduces glucose production, this might improve glucose tolerance after gastrectomy. These results are consistent with a report that GLN supplementation improved insulin’s systemic action, increased muscle glucose uptake, and reduced hepatic glucose production in DIO rats [14]. Our previous study also showed that dietary GLN supplementation increased hepatic glycogen synthesis and reduced gluconeogenesis through regulating the PI3K-Akt pathway in DIO mice complicated with limb ischemia [30]. On the other hand, elevated adiponectin levels observed in the GLN group were considered to have a glucose-lowering effect and improve IR [31,32]. Second, the GLN group showed more-pronounced weight loss at day 3 after the gastrectomy. A previous study reported that rats treated with a GLN-supplemented high-fat diet exhibited improved insulin action and signaling only in the liver and muscles but not in adipose tissues. This tissue-specific IR response prevents adipose mass accumulation and reduces weight gain [15]. A human study also showed that oral GLN supplementation reduced the waist circumference in subjects with overweight and obesity [15]. In addition, adiponectin-induced energy expenditure and fatty acid oxidation may also promote weight loss after surgery [29,30]. Third, GLN administration attenuated adipocyte inflammation and macrophage infiltration after the gastrectomy. Moreover, the inflammatory cytokines produced in the PLF, the primary abdomen injury site, were reduced. A recent in vitro study reported that GLN is the most markedly reduced amino acid in fat tissues obtained from subjects with obesity. Decreased GLN levels in adipose tissues result in increased nuclear O-GlcNAcylation in adipocytes that may consequently activate the transcription of proinflammatory proteins [33]. Our results are consistent with a report that administration of GLN reduced macrophage infiltration and attenuated expressions of proinflammatory genes and proteins in adipocytes [33]. There is a close link between inflammation and IR. Attenuated inflammation may contribute to the improvements in IR and glucose tolerance [34] as observed in the group with GLN administration. Since only 3 days after sleeve gastrectomy was observed in this study, the long-term effects of GLN on obesity with sleeve gastrectomy require further investigation.

## 5. Conclusions

This is the first study to investigate the impacts of intravenous GLN administration on glucose homeostasis and inflammation in DIO mice after bariatric surgery. The results demonstrated that treatment of DIO mice with GLN downregulated hepatic proteomic profiles associated with gluconeogenesis and improved glucose tolerance. Moreover, macrophage infiltration and adipose tissue inflammation were attenuated 1 d and 3 d after the sleeve gastrectomy. The findings provide basic information and imply that GLN may improve glucose tolerance and attenuate inflammation shortly after the bariatric surgery in subjects with obesity.

## Figures and Tables

**Table 1:** Compositions of the high-fat diets.

Ingredients	g/kg
Casein	259.13
L-Cysteine	3.89
Maltodextrin	161.96
Sucrose	89.14
Cellulose	64.78
Soybean oil	32.39
Lard	317.44
Mineral mix ^1^	12.96
Dicalcium phosphate	16.84
Calcium carbonate, 1H_2_O	7.13
Potassium citrate	21.38
Vitamin mix ^2^	12.96
Total	1000

^1^ The compositions of the mineral mixture are listed as following (mg/g): calcium phosphate dibasic, 500; sodium chloride, 74; potassium sulfate, 52; magnesium oxide, 24; potassium citrate monohydrate, 20; manganese carbonate, 3.5; ferric citrate, 6; chromium potassium sulfate, 0.55; zinc carbonate, 1.6; cupric carbonate, 0.3; potassium iodate, 0.01; sodium selenite, 0.01. ^2^ The compositions of the vitamin mixture are listed as following (mg/g): DL-α-tocopherol acetate, 20; nicotinic acid, 3; retinyl palmitate, 1.6; calcium pantothenate, 1.6; pyridoxine hydrochloride, 0.7; thiamin hydrochloride, 0.6; riboflavin, 0.6; cholecalciferol, 0.25; D-biotin, 0.05; menaquinone, 0.005 and cyanocobalamin, 0.001.

**Table 2:** Sequences of oligonucleotide primers used in the PCR amplification.

		Primer Sequences (5′→3′)
GAPDH	Forward	TGCACCACCAACTGCTTAG
	Reverse	GGATGCAGGGATGATGTTC
TNF-α	Forward	AAATGGGCTCCCTCTCATCAGTTC
	Reverse	TCTGCTTGGTGGTTTGCTACGAC
IL-1β	Forward	TGCCACCTTTTGACAGTGATG
	Reverse	ATGTGCTGCTGCGAGATTT
IL-6	Forward	TCCTACCCCAACTTCCAATGCTC
	Reverse	TTGGATGGTCTTGGTCCTTAGCC
CD68	Forward	TGTTCAGCTCCAAGCCCAAA
	Reverse	ACTCGGGCTCTGATGTAGGT
EMR-1	Forward	ACCTTGTGGTCCTAACTCAGTC
	Reverse	ACAAAGCCTGGTTGACAGGTA
MCP-1	Forward	GATTCACATTTGCGCTGCCT
	Reverse	TGAGCCTGGGAGATCACCAT

GAPDH, glyceraldehyde 3-phosphate dehydrogenase; TNF, tumor necrosis factor; IL, interleukin; CD68, cluster of differentiation 68; EMR-1, EGF-like module-containing mucin-like hormone receptor-like 1; MCP-1, macrophage chemoattractant protein-1.

**Table 3:** Plasma adipokine, glucose, and insulin levels after 10 weeks of feeding.

Parameter	NC	HF
Leptin (ng/mL)	4.64 ± 0.85	11.01 ± 1.40 *
Adiponectin (µg/mL)	5.69 ± 0.56	9.98 ± 0.80 *
Glucose (mg/dL)	100.32 ± 20.38	221.47 ± 21.36 *
Insulin (µIU/mL)	18.65 ± 0.12	55.23 ± 1.12 *

Data are expressed as the mean ± SEM. NC, normal control group; HF, high-fat diet group. All data are representative of duplicate measurements (*n* = 8). Differences between groups were analyzed by an unpaired *t*-test. * Significantly differs from the NC group.

**Table 4:** Plasma glucose and insulin levels of the experimental groups.

Group	Glucose (mg/dL)	Insulin (μIU/mL)
SH	213.9 ± 10.6	96.2 ± 27.2
1S	225.5 ± 10.3	88.9 ± 12.7
1G	122.4 ± 12.2 *^,#^	40.6 ± 6.1 *^,#^
3S	189.6 ± 10.1	80.2 ± 8.8
3G	108.2 ± 6.2 *^,#^	42.8 ± 6.6 *^,#^

Data are expressed as the mean ± SEM. SH, sham group; 1S, saline-injected group sacrificed 1 day after the gastrectomy; 1G, GLN-injected group sacrificed 1 day after the gastrectomy; 3S, saline group sacrificed 3 days after the gastrectomy; 3G, GLN-injected group sacrificed 3 days after the gastrectomy. * Significantly differs from the SH group. ^#^ Significantly differs from the S group at the same time point (*p* < 0.05).

**Table 5:** Canonical pathway analysis of hepatic enzyme expressions in the gluconeogenesis pathway between two groups.

Identifier	Enzymes	Description	Log_2_ (3S/3G)	*p* Value
Q91Y97	ALDOB	Aldolase, fructose-bisphosphate B	0.76	0.001
P17182	ENO1	Enolase 1	0.68	0.006
Q9QXD6	FBP1	Fructose-bisphosphatase 1	0.69	0.03
P14152	MDH1	Malate dehydrogenase 1	0.41	0.04
Q05920	PC	Pyruvate carboxylase	1.18	0.03
P16858	GAPDH	Glyceraldehyde-3-phosphate dehydrogenase	1.6	0.03
P17751	TPI1	Triosephosphate isomerase	1.59	0.03

The multiple fold change of protein expressions was uploaded to IPA software to analyze interaction networks of differentially expressed proteins. Significance (*p* value of the overlap) was calculated by Fisher’s exact test; *p* < 0.05 represents a significant difference.
